# Factors influencing medical expenditures in patients with unresolved facial palsy and pharmacoeconomic analysis of upper eyelid lid loading with gold and platinum weights compared to tarsorrhaphy

**DOI:** 10.1186/s13561-024-00506-6

**Published:** 2024-04-27

**Authors:** Izabela Nowak-Gospodarowicz, Marcin Gospodarowicz, Marek Rękas

**Affiliations:** 1 Department of Ophthalmology, Military Institute of Medicine - National Research Institute, 128 Szaserow St, 04-141 Warsaw, Poland; 2 Institute of Banking, Warsaw School of Economics, Niepodległości 162, 02-554 Warsaw, Poland

**Keywords:** Facial palsy, Medical expenditures, Incremental cost-effectiveness ratio (ICER), Cost utility analysis (CUA), Exposure keratopathy, Gold weights, Platinum chains, Tarsorrhaphy, Pharmacoeconomics

## Abstract

There are no standards in diagnostic and therapeutic approaches to eye care in incomplete eyelid closure due to unresolved facial palsy (FP). Loading of the upper eyelid (UELL) with gold weights (GWs) or platinum chains (PCs) is a highly effective procedure for the correction of lagophthalmos. Despite this, the procedure is used infrequently in our country because of the relatively high price of the implant and the lack of reimbursement. The aim of this research was to assess the factors influencing medical expenditures in this group of patients and to analyze utility costs for the UELL procedure with the use of GW and PC compared to tarsorrhaphy.

**Material and methods** The costs of 88 surgical procedures (40 GWs, 11 PCs and 37 tarsorrhaphies) and medical expenditures before and after surgery were calculated based on reporting of materials, staff salaries and the SF-36 questionnaire. Distribution quartiles of the cost per QALY measure (dependent variable) was assessed via an ordered logistic regression model with eight explanatory variables.

**Results** The calculated total cost of the surgery was US$209 for tarsorrhaphy, US$758 for UELL with a GW and US$1,676 for UELL with a PC. Bootstrapped costs per QALY values (CUI) in 88% of cases were below the US$100,000 cutoff. Etiology and duration of facial palsy and presence of Bell’s phenomenon were factors that significantly influenced the CUI. Patient gender and age, history of previous eyelid surgery, and presence of corneal sensation were found to be not significant (*p* > 0.1). Calculated ICER for GW was US$1,241.74/1QALY and ICER for PC was US$13,181.05/1QALY compared to tarsorrhaphy.

**Conclusions** Eye protection in patients with FP should be a crucial element of health policy. Findings suggest UELL procedure with a GW or a PC to be a cost-effective procedure with GW being the most cost-effective.

## Introduction

Facial palsy (FP) is a challenge for specialists in many fields of medicine. Normal function of the facial nerve is necessary for speech, eating, drinking and facial expressions such as surprise, happiness or anger, therefore its damage significantly affects an individual’s private and public quality of life [1–5]. FP affects about 40 individuals per 100,000 annually, but when unresolved (fortunately uncommon at < 1/100,000/year), brings disabling sequelae [7, 8]. Permanent dysfunction of the facial nerve that supplies the orbicularis oculi muscle, which is responsible for closing the eye, is often a complication resulting from the presence of a tumor or surgery performed to remove it. Patients with unresolved FP do not return to social and professional activities, and struggle with the consequences of FP. The eye, devoid of protection from non-closing eyelids, is constantly exposed to external factors. Thus, improvement of facial nerve function becomes the overriding goal in the therapeutic process in order to prevent the development of severe ocular complications leading to corneal ulceration, perforation and loss of the eye [5, 9–12].

There are no standards in diagnostic and therapeutic procedures for eye care in incomplete eyelid closure due to unresolved FP [12–14]. Patients are often referred to an ophthalmologist too late (i.e., with severe ulceration of the cornea, which may result in eye perforation). Generally, the most common treatment in such cases is eyelid suturing (tarsorrhaphy) to protect and heal the surface of the eye. However, suturing is associated with a limited visual field and the cosmetic effect is not acceptable to either the patient or the physician. Loading the upper eyelid with gold weight (GW) or platinum chain (PC) is a safe and effective alternative to this procedure, ensuring not only closing but also opening of the eyes [15, 16]. Despite this, the procedure is used infrequently in our country or often with delay. An obstacle to its use may be the relatively high price of the implant, the lack of reimbursement in some countries and the lack of uniform guidelines developed by interdisciplinary teams to assess, treat or refer a patient with FP.

The aim of this study was to assess the factors influencing medical expenditures in patients with unresolved FP and to conduct a pharmacoeconomic analysis of the upper eyelid lid loading (UELL) procedure with GWs and PCs in the treatment of lagophthalmos compared to tarsorrhaphy.

To our knowledge this is the first pharmacoeconomic study completed to assess medical expenditures and the utility cost of UELL by examining the distribution of the cost per QALY measure. We hope this research will add new insights to our understanding of health care in patients with facial nerve dysfunction.

## Material and methods

This prospective, single-center study was conducted between 2012 and 2021 according to the rules of good medical practice. All methods were performed in accordance with the relevant guidelines and regulations. The study adhered to the Declaration of Helsinki and was approved by the local institutional review board (ethical approval number 57/WIM/2011, received on 17 August 2011). All study participants provided written, informed consent for surgical treatment and participation. Informed consent to publish identifying information/images was obtained from all study participants.

During this time, 64 adult patients (22 men and 42 women, mean age 55 years) with unresolved FP and eye symptoms due to incomplete eyelid closure came to the clinic with a health condition that qualified participation in this study (including a 36 month follow-up). All patients presented with at least grade 4 dysfunction of the facial nerve according to the House Brackman scale [17] and were assessed according to the Sunnybrook Facial Grading System [18]. The inclusion criteria were: (1) Unresolved FP and unchanged lagophthalmos for at least 3 months despite intensive rehabilitation, (2) ocular symptoms reported by the patient due to exposure keratopathy which did not respond to conservative treatment (topical treatment with moisturizers and eye patching or moist chamber), (3) at least fair function (> 4 mm) of the levator muscle of the upper eyelid, and (4) condition of both eyelid skin and orbicularis oculi muscle allowed for surgery. All patients underwent successful UELL procedures (52 with implantation of GWs and 12 with implantation of PCs) to treat incomplete eyelid closure. A detailed description of the study group and surgical techniques performed are described elsewhere [5, 13, 19]. Demographic data, working status and data on the etiology, duration of FP, and history of ophthalmic treatment were obtained from medical history preoperatively.

Each patient completed the validated Polish version of the SF 36v2 quality of life questionnaire twice: before and 6 months after surgery (license No. QM022281, amended to QM023605, to use the SF36 questionnaire was obtained from Quality Metrics Inc.) [20]. Two control groups were selected: a group of 37 patients treated with tarsorrhaphy (TC) in our ward in 2011–2013 who completed the SF-36 questionnaire and a group of 53 healthy individuals (HC). The HC group was recruited between January 2013 and December 2014. After asking the question "Have you ever been treated for any eye diseases?" and obtaining a negative answer, a questionnaire was distributed among administrative employees, orderlies, nurses, doctors, kitchen workers as well as random people in one city park and in the center of our city who agreed to complete the SF-36 questionnaire without being paid. These control groups were recruited in order to compare utility indexes and to estimate medical expenditures per capita per month for healthy individuals in our country.

A shorter survey (the SF-6D utility index) was extracted from the SF-36 questionnaire, as introduced by Brazier et al. [21, 22]. SF-6D focuses on seven of the eight health domains covered in the SF-36v2 questionnaire: physical functioning, performing social roles (combined physical and emotional roles), feeling pain, mental health and vitality. As such, it does not only focus on a narrow view of general health. SF-6D scoring takes into account any limitations in work or other activities due to impaired physical health, limitations due to emotional problems, feelings of pain, nervousness and depression, with the value 0.0 indicating worst possible state of health and 1.0 the best. A computer algorithm for deriving a preference-based index from SF-36 data via the SF-6D (available from the author) was used for both patients (before surgery and 6 months after UELL) and both control groups (TC and HC).

The SF-6D health index method is used internationally for calculating Quality Adjusted Life Years (QALYs) [22, 23]. A QALY is a universal health outcome measure based on patient reported health and is applicable to all individuals and all diseases, thereby enabling comparisons across diseases. QALY is a measure of health benefit resulting from medical interventions. QALY is the sum of the number of years lived after an intervention with a certain quality of life (regarding utility). Utilities are measured on a scale of 0.0 (representing death) to 1.0 (representing perfect health). Some disease conditions are considered worse than death, thus a negative utility value is possible [24].

In the study group, to estimate QALYs preoperatively, a time dimension was assessed with the use of the algorithm described by Kharroubi et al. [25] which included parametric and non-parametric values of SF-6D, SF-6D1 scores and SF-6D2 scores.

Assuming that the utility index associated with non-affected health states corresponds to a value of 1, the parametric SF-6D utility score, representing a patient’s health during the months of documented symptomatic illness, can be transformed into a QALY loss estimate using the following equation:

$$\mathrm{QALY}\;\mathrm{loss}\;\mathrm{SF}-6\mathrm D1\:=\:1\;-\;\lbrack\mathrm{SF}-6\mathrm D1\;\mathrm{score}-(12-\;\mathrm{months}\;\mathrm{of}\;\mathrm{illness})/12\rbrack.$$

QALY loss from the same survey but using a more recent non-parametric method used by other authors was calculated in a similar manner: QALY loss SF-6D2 = 1 – [SF-6D2 score-(12- months of illness)/12] [23, 25].

Both indicators (SF-6D1 and SF-6D2) were compared using correlation measures and statistical tests. A Wilcoxon test showed that they both carry the same information load, hence further analyses covered only the non-parametric version of the measure, as suggested by Kharroubi et al. [25].

The QALY gain in patients with FP was estimated as the differences between QALY values before and within 6 months after surgery according to the following equation:

$$\mathrm{The}\;\mathrm\Delta\;\mathrm{QALY}\:=\:1-\mathrm{QALY}\;\mathrm{Loss}\;\mathrm{SF}-6\mathrm D2$$

The estimated medical expenditures per patient per month in patients with FP before and 6 months after surgical treatment was estimated on the basis of the SF-36 questionnaire as introduced by Brazier et al. and adopted by the Department of Health and the National Institute for Health and Care Excellence (NICE) [21, 26, 27]. The following parameters were analyzed for SF-36v2: gender, age, total physical health component score, and total mental health component score. In the area of physical health, the questions in the SF-36 questionnaire focused on: physical functioning, pain perception, and level of professional activity. In the area of mental health, questions focused on: performing social functions, professional activity, energy level and subjective perception of illness. Results were compared with the normative results of the SF-36 questionnaire database obtained from an average person of the same sex and age as well as with a statistically healthy person in the United Kingdom.

Estimated costs were analyzed in detail by sex and age and were compared between the test and control groups. Costs of the UELL surgical procedures were calculated from the service provider’s point of view and were based on reports on the use of materials in the operating room as well as drugs and dressings during hospital stay (calculated based on purchase prices according to the price list at our clinic in years 2018–2021) [28, 29]. Costs of tarsorrhaphy were estimated based on the valuation of the procedure in our country (ICD-9 code). The Cost -Utility Index (CUI) was calculated for each surgical procedure according to the formula:

Cost-utility ratio = Cost_A_ − Cost_B_/QALY_A_ − QALY_B_ [30, 31].

Cost A = estimated medical expenditures per capita per month in patients with FP before surgical treatment.

Cost B = estimated medical expenditures per capita per month in patients with FP after surgical treatment + calculated cost of surgery.

QALY A = estimated QALY in patients with FP before surgical treatment.

QALY B = estimated QALY in patients with FP after treatment.

To provide information on the comparative cost-effectiveness of 3 treatment strategies ( tarsorrhaphy, UELL with GW and UELL with PC) the following incremental cost-effectiveness ratios (ICER) were calculated: for UELL with GW (more expensive therapy) vs tarsorrhaphy (the alternative) and for UELL with PC (more expensive therapy) vs tarsorrhaphy (the alternative).

The ICER was calculated in the units US$/QALY as the measure of effectiveness [30, 32, 33].

Interventions were ordered from least costly to most costly and in increasing order of effectiveness.

The alternatives were ordered from lowest ICER to highest.

Statistical analysis was performed using SPSS software (IBM Corp. Released 2012. IBM SPSS Statistics for Windows, Version 21.0. Armonk, NY: IBM Corp, USA). The Kolomogorov Smirnov test and the Shapiro Wilk test were used to check the normality of the distribution. To compare two dependent groups the Wilcoxon rank test was performed. The U Mann–Whitney test was performed to compare two independent groups. The *p* value < 0.05 was considered statistically significant. For Health Related Quality of Life (HRQoL)-based measures, the literature suggests the use of a nonparametric approaches to determine the true parameters of the sample distribution (mean value, standard deviation, and confidence intervals). For this purpose, a nonparametric bootstrapping approach was used for 10,000 iterations [34]. In order to assess factors influencing the medical expenditures in patients with unresolved FP, an ordered logistic regression model was built.

An ordered logistic regression model was used with the dependent variable described by the quartiles of the distribution of the cost per QALY measure. The dependent variable was comprised of four values (1, 2, 3, 4) describing each quartile of the distribution. The value 1 represented the lowest (25%) quartile of the distribution (i.e. the lowest costs), while the value 4 represented the highest (100%) quartile of the distribution (i.e. the highest costs). Eight explanatory variables of varying nature (continuous and binary) were used. Continuous variables included duration of FP, patient's age, Sunnybrook facial grading system rating, and binary variables including the presence of Bell's phenomenon, presence of corneal sensation reflecting intact blink reflex, the etiology of FP (in the sense of the presence of Cerebellopontine Angle (CPA) tumor), as well as the patient's gender. Regression coefficient values, odds ratio measure, and marginal effect indices were used to assess the direction and strength of the relationship between the variables.

## Results

**Fig. 1:**
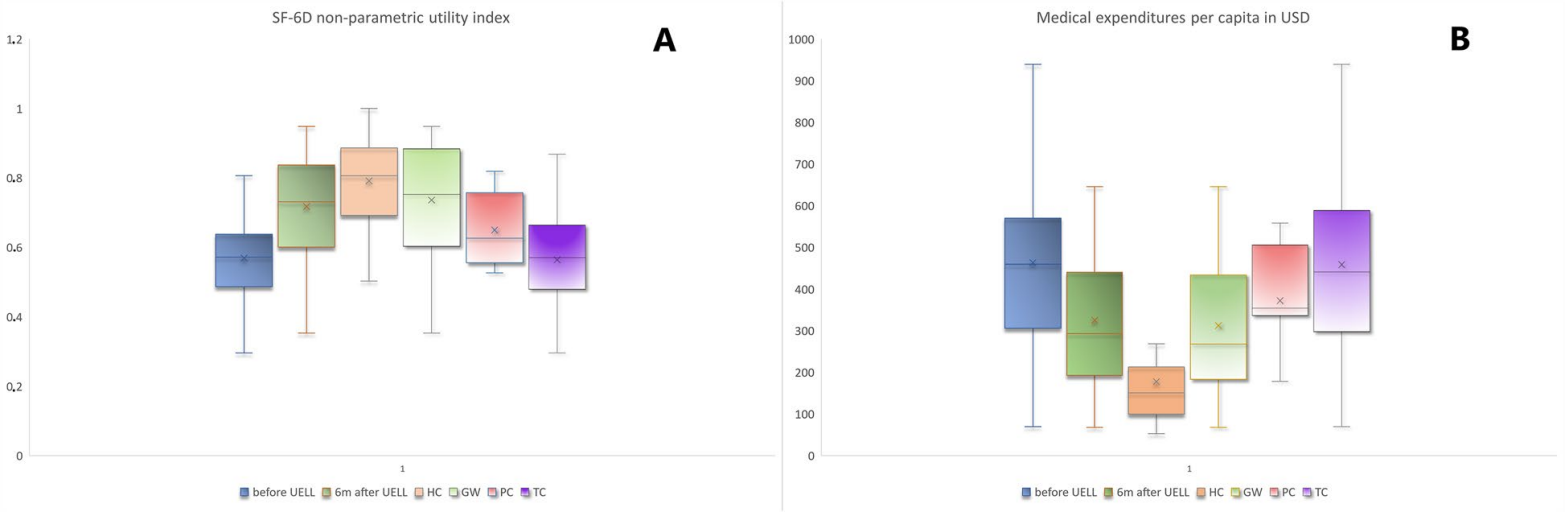
Box plot – non parametric utility index (SF-6D) and medical expenditures per capita per month in USD. UELL Upper Eyelid Lid Loading, HC Control Group of Healthy Individuals, TC Control Group of Patients treated with Tarsorrhaphy, GW Patients treated with UELL with a Gold Weight, PC Patients treated with UELL with a Platinum Chain

Completed questionnaires (229 total) were obtained from the patients with FP (51 out of 64 individuals treated with UELL: 37 females, 14 males with a mean age 55 ± 17 years and 37 out of 42 individuals treated with tarsorrhaphy: 27 females, 10 males with a mean age 51 ± 16 years) before and 6 months after surgery and from healthy individuals (53 total, 27 females and 26 males with an age range or 19–68, mean age 42). Among the study group, 40 (78%) patients had FP due to CPA tumor, 4 (8%) after parotid gland tumor surgery, 3 (6%) after trauma, 2 (4%) with unresolved Bell’s palsy, and 2 (4%) with congenital FP. The average Sunnybrook score in the FP group was 27.9 ± 13 in the GW group and 28.1 ± 12.6 in the PC group and 28.3 ± 11 in the TC group (*p* > 0.1). The mean time between the onset of FP and surgical intervention for correction of lagophthalmos was 116 ± 202 months. Before surgical treatment all patients were professionally inactive mostly for health reasons (34 patients, 66.7%) or because of age (17 patients, 33.3%). Lagophthalmos was on average 7 ± 3 mm in the GW group and 8 ± 4 mm in the PC group and 7.5 ± 4 mm in the TC group prior to surgery (*p* > 0.1). Poor Bell’s phenomenon was observed in 9 (18%) patients. The presence of corneal sensation reflecting intact blink reflex was noted in 34 (66.7%) patients. Patients instilled on average 9 ± 6 drops of moisturizing drugs per day. Exposure keratopathy with no improvement after conservative treatment was the main reason surgery was performed on all patients in the FP group. Significant improvement of exposure keratopathy and visual acuity was noted in all patients after a successful UELL procedure [5, 13, 19]. The average weight of the implant was 1.6 ± 0.2 g in the GW group and 1.6 ± 0.4 mm in the PC group (*p* > 0.1). Return to professional activity was noted in 17 patients (33.3%) at the 6 month follow-up. The lowest SF-6D values were noted in the group of patients with FP before UELL. The highest SF-6D was noted in the group of healthy individuals. This was consistent with the observed mean medical expenditures per person per month in these groups. The differences between groups were statistically significant (*p* < 0.0001) (Fig. 1).

The calculated total cost of the UELL surgical procedure was PLN 6,167.53 for a PC and PLN 2,786.53 for a GW (which according to an exchange rate of US$ 1 = 3.68 PLN, as of June 2018) totals US$1,676.78 and US$758.03 respectively. Utility indices increased significantly (*p* < 0.0001) (Fig. 1A), and ME decreased on average by US$137.77 ± 94.83 / month after surgery (*p* < 0.0001, Fig. 1B). However, differences in estimated costs remained significantly higher in postoperative patients compared to healthy individuals (*p* = 0.0168). The cost of tarsorrhaphy was PLN 769 (US$ 208.97).

**Fig. 2:**
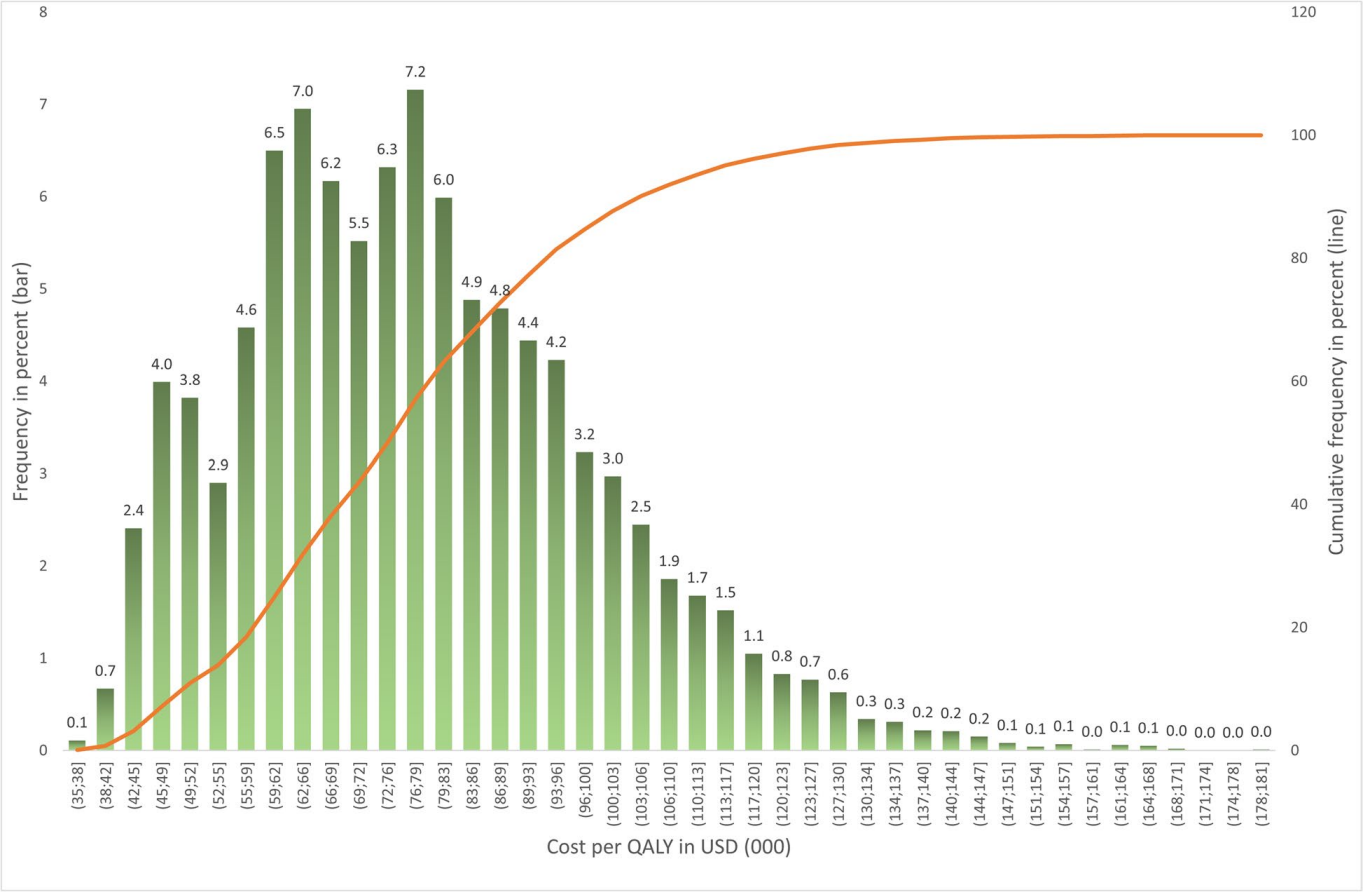
Histogram of bootstrapped values of Cost per QALY in USD (000)

Estimated CUI for UELL in the study group was about US$ 77,000 for GW, US$116,000 for PC and US$ 68,000 for the TC group. Bootstrapping approach showed that nearly 88% of costs per QALY value were below the US$10,0000 cutoff. The mean value for the estimated sample was US$77,476 (Fig. 2).

The estimation results are collected in Table 1.

**Table 1:** Ordinal logistic regression model (dependent variable cost-utility – four quartiles of distribution)

Type	Variable	Coeff.	*P*-val	Sig.	Odds ratio	Marginal effects											
						Q1			Q2			Q3			Q4		
						dx/dy	*P*-val	Sig.	dx/dy	*P*-val	Sig.	dx/dy	*P*-val	Sig.	dx/dy	*P*-val	Sig.
Continous (months)	Duration of the FP	0.007 *(0.002)*	0.02	**	1.01	-0.00 *(0.00)*	0.02	**	-0.002 *(0.00)*	0.62		-0.002 *(0.00)*	0.77		0.002 *(0.001)*	0.02	**
Continous (years)	Patient's age	-0.02 *(0.03)*	0.57		0.98	0.00 (0.01)	0.57		0.00 (0.01)	0.70		0.00 (0.01)	0.80		-0.004 *(0.007)*	0.57	
Binary (0–1)	Absence of Bell’s phenomenon	3.756 *(1.41)*	0.01	***	42.7	-0.92 *(0.35)*	0.01	***	-0.018 *(0.03)*	0.62		0.015 *(0.05)*	0.77		0.923 *(0.348)*	0.01	***
Binary (0–1)	Absence of corneal sensation	0.861 *(0.8)*	0.28		2.37	-0.21 *(0.20)*	0.28		-0.004 *(0.09)*	0.64		0.003 *(0.01)*	0.78		0.211 *(0.196)*	0.28	
Binary (0–1)	Presence of the previous eyelid surgery	*0.260* *(0.78)*	0.74		1.3	-0.06 *(0.19)*	0.74		-0.001 *(0.004)*	0.78		0.001 *(0.05)*	0.83		0.064 *(0.192)*	0.74	
Binary (0–1)	Etiology of the FP (CPAT)	2.726 *(1.43)*	0.06	*	15.3	-0.67	0.06	*	-0.013	0.62		0.011	0.77		0.670	0.06	*
Binary (0–1)	Patient's gender (men)	4.213 *(3.72)*	0.26		67.5	-1.03 *(0.91)*	0.26		-0.020 *(0.04)*	0.65		0.017 *(0.06)*	0.77		1.035 *(0.917)*	0.26	
Continous (pcts.)	Sunnybrook Facial Grading System	-0.17 *(0.6)*	0.17		0.85	0.04 *(0.01)*	0.17		0.001 *(0.001)*	0.69		-0.001 *(0.001)*	0.77		-0.041 *(0.015)*	0.17	
Threshold	(1- > 2)	-14.8 *(20.6)*	0.47														
Threshold	(2- > 3)	-14.6 *(20.6)*	0.48														
Threshold	(3- > 4)	-14.3 *(20.6)*	0.49														
Model statistics	Log-Likelihood:		-41.23														
	AIC:		110.47														
	McFadden's R^2^:		0.20														

Standard errors in parentheses, # of obs. 51

Significance level: ***—1%, **—5%, *—10%

**Fig. 3:**
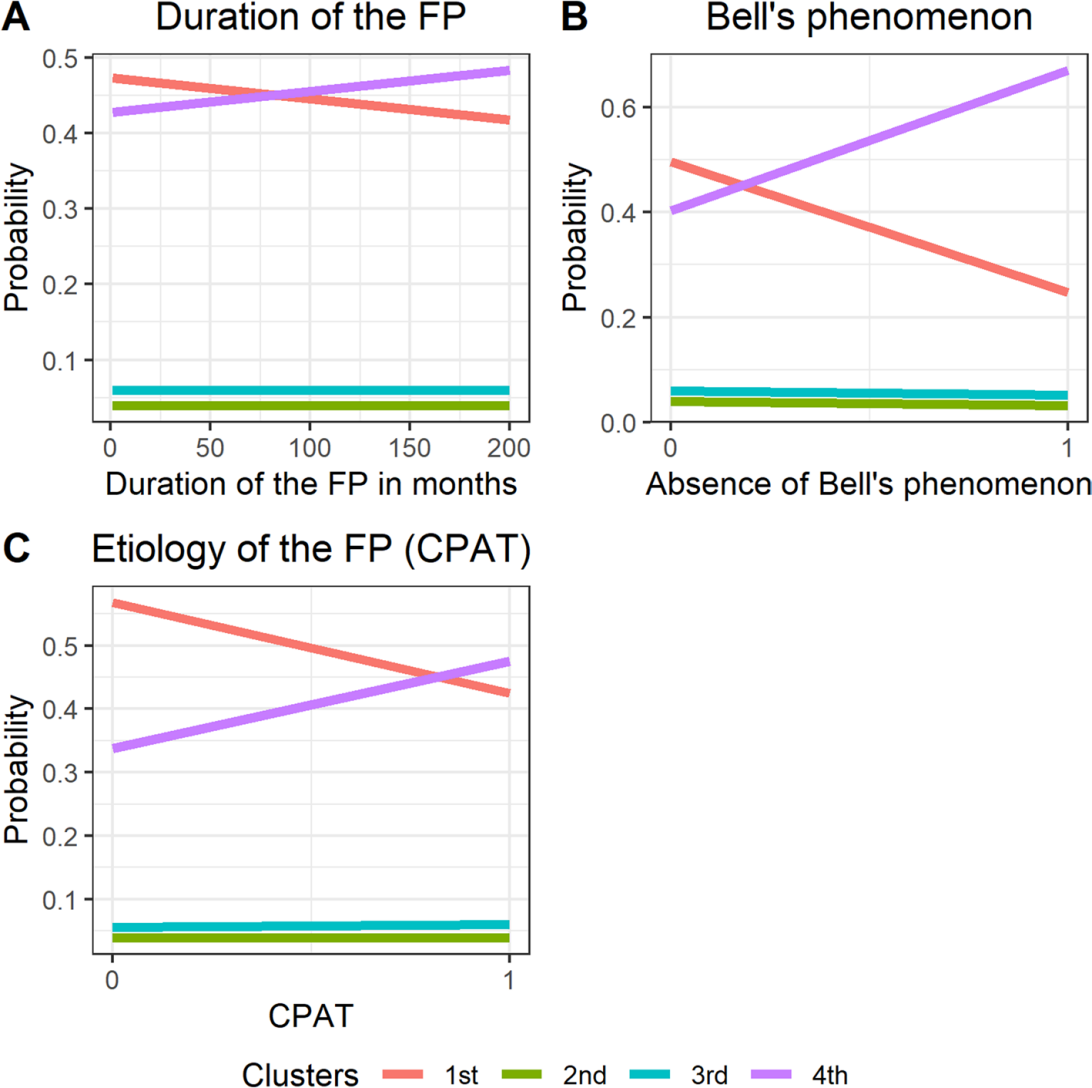
Results of the multinomial ordered logit model for Cost per QALY with statistically significant independent variable: (**A**)—duration of the FP, (**B**)—Bell’s phenomenon, (**C**)—Etiology of the FP (CPAT), assuming 4 graded levels (quartiles)

Etiology and duration of FP and presence of Bell’s phenomenon were the factors that significantly influenced CUI. Longer duration of symptoms prior to surgery and CPA tumor (as the etiology of FP) were connected with an increased probability of higher cost per 1 QALY gain in the study group (Fig. 3A, B). In turn, preserved Bell’s phenomenon was connected with an increased probability of lower cost per QALY gain in the study group (Fig. 3C).

Patient gender, age, history of previous eyelid surgery, and presence of corneal sensation were found not to be significant factors influencing the CUI in patients with ocular complications due to unresolved FP (*P* > 0.1). An ICER calculated for UELL with a GW (more expensive therapy) vs tarsorrhaphy (the alternative) was $ 1241.74/1QALY. An ICER calculated for UELL with a PC (more expensive therapy) vs tarsorrhaphy (the alternative) was $13181.05/1QALY.

## Discussion

Cost-utility analysis in medicine compares the costs of medical procedures, expressed in monetary units, with the clinical benefits, calculated as QALY units [30–33]. An intervention is typically thought to be very cost-effective if its cost–utility ratio is < US$50,000/QALY and cost-effective if it costs is < US$100,000/QALY. World Health Organization (WHO) standards suggest < US$40,000/QALY as very cost-effective and < US$12,0000/QALY as cost-effective [31, 35–37]. This study shows that for every US$77,000 invested in UELL with the use of a GW and US$116,000 invested in UELL with a PC to treat ocular complications due to unresolved FP, we would expect to observe 1 additional perfect year of health gained (1 QALY) as compared to treating the same population conservatively. According to the WHO, UELL with the use of a GW or a PC appears to be a cost-effective procedure with a bootstrapped 88% cost per QALY value below US$100,000 [31–37] (Fig. 2). The results indicate that a statistically significant relationship exists with three characteristics—duration of FP symptoms, absence of Bell's phenomenon and etiology of FP (i.e., the source of paralysis based on tumor type). An increase in the duration of FP is associated with increased costs invested to QALY gain.

This cost would be even higher if time costs related to seeking or obtaining the intervention were included. This duration, which in our group was 116 ± 202 months, has an opportunity cost in that one cannot produce or consume in other areas so, in theory, it should be valued and included. This long duration, related to seeking effective treatment, suggests a gap in our healthcare system in which there are no preventive programs nor procedures dedicated to treat ocular complications of long-lasting lagophthalmos financed from the state budget. Surprisingly, the lack of comprehensive multidisciplinary care is manifest even in the health care systems with financing of UELL from the state budget in the EU countries: Germany, Netherlands, United Kingdom and Swiss [38–41]. This is why we were trying to identify factors influencing medical expenditures in patients with unresolved FP.

The absence of Bell's phenomenon and etiology of FP (specifically CPA tumor) also significantly increased estimated costs. For these two variables, the strength of the relationship described by the odds-ratio is very high (Table 1). Bell’s phenomenon, which is responsible for upward rotation of the eyeballs when closing the eye, has a protective effect on the surface of the eye. Our previous study shows that good Bell’s phenomenon is a positive predictive factor that significantly influences visual function of the eye in patients with unresolved FP [13]. Therefore we propose that it should be assessed in every patient with FP. Poor Bell's phenomenon, in our opinion, should lead to immediate referral to an ophthalmologist in order to protect the eye and reduce the cost of medical care of patients with FP [13, 14]. CPA tumor was the cause of FP in 78% of patients in our study. Significantly higher costs in this group were probably connected with concomitant dysfunction of the other cranial nerves resulting from the anatomy of CPA.

In the case of marginal effects, we note that for statistically significant variables, (e.g. the duration of FP), a delay in treatment by UELL by one unit (month) results in a decreased probability that the costs for a given patient are in the lowest quartile of costs distribution (and, further, an increased probability that the costs for a given patient are in the highest quartile). For the other variables and their marginal effects, the absence of Bell's phenomenon decreases the probability that costs will be in the lowest quartile of the distribution and increases the probability that a patient's cost will be in the highest quartile. In the case of the etiology of FP, if it is caused by a CPA tumor, the probability that the costs are in the lowest quartile decreases and the probability that the patient costs are in the highest quartile increases. These relationships are illustrated in Fig. 3, which shows the univariate relationship between statistically significant explanatory variables and the dependent variable.

The analysis of other variables, while not significant, suggest some logistic trends, which may indicate a direction of further research and health policy (especially with larger groups of patients). A higher Sunny Brook score and preserved corneal sensation (reflecting an intact blink reflex) positively influenced cost per QALY gain, which means lower cost per QALY gain. In turn, a history of previous eyelid surgery and gender (female) were connected with a higher cost per QALY gain in the study group.

The idea to fill in the noted health system policy gap in our country with UELL for the treatment of lagophthalmos assumes that such a procedure be effective, safe, relatively easy, cost-effective and reversible. Based on our experience and other studies [5, 10–16, 19] we conclude that UELL may fulfill these assumptions. The cost of tarsorrhaphy was PLN 769 (US$208.97) and was at least three times lower than UELL procedures. Taking into account ICER calculations, tarsorrhaphy was the cheapest treatment option, but with significantly lower QALY in comparison with QALY after UELL procedures (Fig. 1). The most cost-effective alternative was UELL with a GW, which had an ICER of US$1,241.74 per QALY gain compared to tarsorrhaphy. An ICER for UELL with a PC was $13,181.05 per QALY, which means that in the latter case (PC) it is necessary to pay over 9 × more per 1 QALY gained than in the case of a GW. A GW is therefore much more cost-effective than a PC with comparable outcomes achieved at a much lower cost.

It should be emphasized that the results obtained are typical in the case of introducing new products or technologies. Research by Szczepura et al. estimates that annual expenditure on hospital treatment for patients with FP in the UK is currently > US$106 mil. Patients with permanent FP can suffer a loss of up to 2 QALYs [41]. PCs are modern and naturally more expensive. It is always the decision of the service provider who, depending on budget, should decide whether it is worth paying more. Fortunately, according to research conducted by Su et al., society perceives the repair of facial paralysis to be a high-value intervention [40].

Complications related to UELL are relatively rare and include weight extrusion, migration, bulging, induced astigmatism responsible for deterioration of visual acuity, under- or overcorrection, presumed allergic reaction to gold and unsatisfactory cosmesis [14–16, 19, 42]*.* Complications that required repeated surgery were noted in 9 patients (14%) in the study group and included: 5 implant extrusions, 2 presumed allergies to gold, 1 excessive ptosis, and 1 unsatisfactory cosmesis [19, 42]. Considering that this gives a percentage of about 1 complication per year, and that the main cost of the procedure is generated from the implant itself (US$428 from a total US$758 for GWs or US$1,345 from a total US$1,676 for PCs), the global cost of treatment of complications related to the procedure is negligible.

This study shows that UELL procedure with implantation of a GW or a PC is a cost -effective procedure for the treatment of ocular complications due to unresolved FP with positive, measurable results in one third of patients who returned to professional activity only 6 months after surgery.

As with any research, our study has some limitations.

Firstly, there is a lack of randomization and differences in the sample size of our groups, which resulted from the lack of financing UELL procedures from the state budget in our country. Considering that irreversible FP is a rare disease and that there is a gap in our health care system in the treatment of ocular complications secondary to FP, this study included the majority of patients with this condition in our country. Moreover the bootstrapping method used in our study allowed us to determine the actual parameters of the distribution and to show the general trend. Secondly, costs of the procedure were calculated according to the standards in one particular clinic, while the monthly cost of medical care was estimated using the modelling method adopted for this purpose in the United States [21, 27]. The calculated costs of the surgical procedure would likely differ if the surgeries had been performed in another country (i.e., in the United States). While the prices for the implant, sutures, and other materials necessary for the procedure are rather uniform, staff compensation and depreciation costs may not be the same. We can assume that the cost effectiveness ratios expressed as a CUI or an ICER should be similar. Given that the method used in our study has been adopted for the assessment of cost effectiveness by health services and regulatory agencies around the world (including Australia, Canada, China, Scotland, Netherlands and Norway) [21, 43, 44], we believe our pioneering calculations appear to be sufficient to influence the direction of health policy for this particular group of patients in many countries. We hope our calculations will inspire the creation of multidisciplinary teams of specialists to deal with the consequences of unresolved FP to optimize patient care with respect to physical condition, quality of life, and socioeconomic factors during the course of a patient’s life.

## Conclusions

This study shows that ensuring the protection of the eye in patients with FP should be an important element of health policy. Data suggests UELL with implantation of a GW or a PC is cost-effective and brings a double benefit: for the health care system, (which saves on the treatment of complications resulting from damage to the unprotected eye) and for patients (who benefit from an improved quality of life). UELL with GWs seems to be the most cost-effective procedure. There is a need to create a clear diagnostic and therapeutic policy for patients with ocular complications due to irreversible FP in our country and many other countries in order to prevent irreparable visual impairment. Among patients suffering from FP, quick and effective ophthalmological care should be guaranteed for patients with poor Bell’s phenomenon and lagophthalmos, especially in whom a CPA tumor was the cause of FP. There is a need to establish multidisciplinary teams of experts in order to formulate uniform guidelines for the treatment of patients with FP and clear reimbursement criteria.

## Data Availability

The data that support the findings of this study are available from the corresponding author upon reasonable request.

## Appendix1 [44]

CHEERS 2022 Checklist

**Table:**

	**Item**	**Guidance for Reporting**	**Reported in section**
**TITLE**			
Title	1	Identify the study as an economic evaluation and specify the interventions being compared.	Title
**ABSTRACT**			
Abstract	2	Provide a structured summary that highlights context, key methods, results and alternative analyses.	Abstract
**INTRODUCTION**			
Background and objectives	3	Give the context for the study, the study question and its practical relevance for decision making in policy or practice.	Introduction
**METHODS**			
Health economic analysis plan	4	Indicate whether a health economic analysis plan was developed and where available.	Methods
Study population	5	Describe characteristics of the study population (such as age range, demographics, socioeconomic, or clinical characteristics).	Methods and results
Setting and location	6	Provide relevant contextual information that may influence findings.	Methods
Comparators	7	Describe the interventions or strategies being compared and why chosen.	Methods
Perspective	8	State the perspective(s) adopted by the study and why chosen.	Methods
Time horizon	9	State the time horizon for the study and why appropriate.	Methods
Discount rate	10	Report the discount rate(s) and reason chosen.	NA
Selection of outcomes	11	Describe what outcomes were used as the measure(s) of benefit(s) and harm(s).	Methods
Measurement of outcomes	12	Describe how outcomes used to capture benefit(s) and harm(s) were measured.	Methods
Valuation of outcomes	13	Describe the population and methods used to measure and value outcomes.	Methods
Measurement and valuation of resources and costs	14	Describe how costs were valued.	Methods
Currency, price date, and conversion	15	Report the dates of the estimated resource quantities and unit costs, plus the currency and year of conversion.	Methods
Rationale and description of model	16	If modelling is used, describe in detail and why used. Report if the model is publicly available and where it can be accessed.	Methods
Analytics and assumptions	17	Describe any methods for analysing or statistically transforming data, any extrapolation methods, and approaches for validating any model used.	Methods
Characterizing heterogeneity	18	Describe any methods used for estimating how the results of the study vary for sub-groups.	Methods
Characterizing distributional effects	19	Describe how impacts are distributed across different individuals or adjustments made to reflect priority populations.	Methods
Characterizing uncertainty	20	Describe methods to characterize any sources of uncertainty in the analysis.	Methods and study limitations
Approach to engagement with patients and others affected by the study	21	Describe any approaches to engage patients or service recipients, the general public, communities, or stakeholders (e.g., clinicians or payers) in the design of the study.	Methods
**RESULTS**			
Study parameters	22	Report all analytic inputs (e.g., values, ranges, references) including uncertainty or distributional assumptions.	Results
Summary of main results	23	Report the mean values for the main categories of costs and outcomes of interest and summarise them in the most appropriate overall measure.	Results and discussion
Effect of uncertainty	24	Describe how uncertainty about analytic judgments, inputs, or projections affect findings. Report the effect of choice of discount rate and time horizon, if applicable.	Results and discussion
Effect of engagement with patients and others affected by the study	25	Report on any difference patient/service recipient, general public, community, or stakeholder involvement made to the approach or findings of the study.	Results and discussion
**DISCUSSION**			
Study findings, limitations, generalizability, and current knowledge	26	Report key findings, limitations, ethical or equity considerations not captured, and how these could impact patients, policy, or practice.	Discussion
**OTHER RELEVANT INFORMATION**			
Source of funding	27	Describe how the study was funded and any role of the funder in the identification, design, conduct, and reporting of the analysis.	Title page
Conflicts of interest	28	Report authors conflicts of interest according to journal or International Committee of Medical Journal Editors requirements.	Title page

## Abbreviations

CPA: Cerebellopontine angle
CUI: Costutility index
EU: European Union
FP: Facial palsy
GW: Gold weight
HC: “Healthy” control
HRQoL: Health Related Quality of Life
ME: Medical expenditures
NICE: The National Institute for Health and Care Excellence
PC: Platinum chain
SF-36: 36-Item Short Form Survey Instrument
SF-6D: The six-dimensional health state short form
TC: Tarsorrhaphy control
QALY: Quality adjusted life year
UK: United Kingdom
UELL: Upper eyelid lid loading
WHO: World Health Organization
